# Evidence of Neutralizing and Non-Neutralizing Anti-Glucosaminidase Antibodies in Patients With *S. Aureus* Osteomyelitis and Their Association With Clinical Outcome Following Surgery in a Clinical Pilot

**DOI:** 10.3389/fcimb.2022.876898

**Published:** 2022-07-18

**Authors:** Shardulendra Prasad Sherchand, Rajan P. Adhikari, Gowrishankar Muthukrishnan, Tulasikumari Kanipakala, John R. Owen, Chao Xie, M. Javad Aman, Richard A. Proctor, Edward M. Schwarz, Stephen L. Kates

**Affiliations:** ^1^ Integrated BioTherapeutics, Inc., Rockville, MD, United States; ^2^ Center for Musculoskeletal Research, University of Rochester Medical Center, Rochester, NY, United States; ^3^ Department of Orthopaedic Surgery, Virginia Commonwealth University, Richmond, VA, United States; ^4^ Departments of Medical Microbiology/Immunology and Medicine, University of Wisconsin School of Medicine and Public Health, Madison, WI, United States

**Keywords:** *Staphylococcus aureus*, osteomyelitis, glucosaminidase, antibodies, immunoassay

## Abstract

*Staphylococcus aureus* osteomyelitis remains a very challenging condition; recent clinical studies have shown infection control rates following surgery/antibiotics to be ~60%. Additionally, prior efforts to produce an effective *S. aureus* vaccine have failed, in part due to lack of knowledge of protective immunity. Previously, we demonstrated that anti-glucosaminidase (Gmd) antibodies are protective in animal models but found that only 6.7% of culture-confirmed *S. aureus* osteomyelitis patients in the AO Clinical Priority Program (AO-CPP) Registry had basal serum levels (>10 ng/ml) of anti-Gmd at the time of surgery (baseline). We identified a small subset of patients with high levels of anti-Gmd antibodies and adverse outcomes following surgery, not explained by Ig class switching to non-functional isotypes. Here, we aimed to test the hypothesis that clinical cure following surgery is associated with anti-Gmd neutralizing antibodies in serum. Therefore, we first optimized an *in vitro* assay that quantifies recombinant Gmd lysis of the *M. luteus* cell wall and used it to demonstrate the 50% neutralizing concentration (NC_50_) of a humanized anti-Gmd mAb (TPH-101) to be ~15.6 μg/ml. We also demonstrated that human serum deficient in anti-Gmd antibodies can be complemented by TPH-101 to achieve the same dose-dependent Gmd neutralizing activity as purified TPH-101. Finally, we assessed the anti-Gmd physical titer and neutralizing activity in sera from 11 patients in the AO-CPP Registry, who were characterized into four groups *post-hoc*. Group 1 patients (n=3) had high anti-Gmd physical and neutralizing titers at baseline that decreased with clinical cure of the infection over time. Group 2 patients (n=3) had undetectable anti-Gmd antibodies throughout the study and adverse outcomes. Group 3 (n=3) had high titers +/− neutralizing anti-Gmd at baseline with adverse outcomes. Group 4 (n=2) had low titers of non-neutralizing anti-Gmd at baseline with delayed high titers and adverse outcomes. Collectively, these findings demonstrate that both neutralizing and non-neutralizing anti-Gmd antibodies exist in *S. aureus* osteomyelitis patients and that screening for these antibodies could have a value for identifying patients in need of passive immunization prior to surgery. Future prospective studies to test the prognostic value of anti-Gmd antibodies to assess the potential of passive immunization with TPH-101 are warranted.

## Introduction

Osteomyelitis is the bane of orthopedic surgery, and there is a great need for novel interventions (Schwarz et al., 2019). Most severe cases involve *Staphylococcus aureus* (Darouiche, 2004), primarily methicillin-resistant *S. aureus* (MRSA) in some regions (Kaplan, 2014), and multidrug-resistant strains are emerging (Assis et al., 2017). Thus, there is a great need for non-antibiotic immune-based approaches to treat these deep infections, as loss of the few remaining antibiotics due to drug resistance is a serious public health threat (Miller et al., 2019). Sadly, infection rates following total joint replacement and trauma surgery have remained largely unchanged over the last 50 years (Schwarz et al., 2019). This is not due to lapses in technique, as adherence to rigorous prophylactic and surgical protocols [e.g., Surgical Care Improvement Project (SCIP) (Stulberg et al., 2010)] failed to reduce infection rates for elective surgery below 1%–2% (Cram et al., 2012). Based on this, the field has concluded that host factors represent an essential role in orthopedic infections (Ricciardi et al., 2020).

*Staphylococcus aureus* osteomyelitis results from various pathogenic mechanisms of immune evasion (Masters et al., 2019; Muthukrishnan et al., 2019). These mechanisms include (1) biofilm formation on the implant (Nishitani et al., 2015) and necrotic bone (Lew and Waldvogel, 2004; Birt et al., 2017), (2) generation of staphylococcal abscess communities in soft tissues and bone marrow (Cheng et al., 2009; Varrone et al., 2014; Yokogawa et al., 2018), (3) intracellular infection including “Trojan horse” macrophages (Masters et al., 2019; Nishitani et al., 2020), and (4) the ability to colonize the osteocytic-canalicular network of live cortical bone (de Mesy Bentley et al., 2017; de Mesy Bentley et al., 2018). As a result, persistence of infection following surgery for *S. aureus* osteomyelitis is common (15%–40%) and often requires multiple surgeries (Salgado et al., 2007; Azzam et al., 2009; Ferry et al., 2009; Ghanem et al., 2009; Parvizi et al., 2009).

Regrettably, 19 *S. aureus* immunizations have been evaluated in Food and Drug Administration (FDA) registration trials, and all failed to demonstrate efficacy (Proctor, 2015; Miller et al., 2019). Acknowledged reasons for these failures include the inability to predict the protective role of staphylococcal immune responses in humans based on animal data (Proctor, 2015; Miller et al., 2019). Thus, we aimed to develop an immunotherapy based on osteomyelitis epidemiology data and monoclonal antibodies (mAb) that have dual-acting mechanisms of action: (1) direct inhibition of critical *S. aureus* enzymes and (2) immunomodulatory activity to stimulate the host response and bacterial clearance (Varrone et al., 2011; Varrone et al., 2014). Based on results in a murine tibial osteomyelitis model that recapitulates several features of implant-associated osteomyelitis (Li et al., 2008), we identified the glucosaminidase (Gmd) protein subunit of *S. aureus* autolysin (Atl) as our lead target for passive immunization (Varrone et al., 2011; Gedbjerg et al., 2013; Varrone et al., 2014; Yokogawa et al., 2018). Of note, other groups also identified Atl as an immunodominant and protective antigen in various animal models (Holtfreter et al., 2010; Brady et al., 2011; Gotz et al., 2014). Atl is also known to be critical for cell wall biosynthesis and degradation during binary fission (Oshida et al., 1995; Sugai et al., 1995; Yamada et al., 1996) and functions as an adhesin (Heilmann et al., 2005) and a biofilm enzyme (Brady et al., 2006), and facilitates host cellular internalization/immune evasion (Hirschhausen et al., 2010). Of the various surface proteins we investigated, only deletion of Atl results in a defective cell division phenotype *in vitro* (Masters et al., 2021). Most importantly, it has been shown that anti-Gmd passive immunization synergizes with vancomycin therapy in rabbit and murine models of infection (Brady et al., 2011; Yokogawa et al., 2018; Kalali et al., 2018). Moreover, our clinical studies of patients with osteomyelitis from prosthetic joint infection (PJI), trauma, and diabetic foot ulcers have found anti-Gmd antibodies in patients that recover from these serious infections (Gedbjerg et al., 2013; Nishitani et al., 2015; Oh et al., 2018). Hence, anti-Gmd antibodies might be a long sought-after biomarker of protective immunity against *S. aureus* (Miller et al., 2019).

In our initial screening for candidates, we utilized an *in vitro Micrococcus luteus* cell wall digestion assay to identify anti-Gmd mAb that inhibits recombinant enzyme activity (Gedbjerg et al., 2013). The results showed that mAb can be either neutralizing or non-neutralizing and that most neutralizing mAb bind to the R3 domain of Gmd (Varrone et al., 2011; Varrone et al., 2014). Based on this initial *in vitro* and *in vivo* research, we derived a mouse IgG1 anti-Gmd mAb (1C11) with high affinity and 1:1 stochiometric neutralizing activity (Gedbjerg et al., 2013; Varrone et al., 2014). We also showed that 1C11 mediates *S. aureus* megacluster formation and opsonophagocytosis *in vitro* (Varrone et al., 2011; Varrone et al., 2014) and had favorable safety and pharmacokinetics in a sheep model of passive immunization (Lee et al., 2020).

We also completed several clinical studies to assess endogenous human anti-Gmd antibodies in osteomyelitis patients and healthy controls (Gedbjerg et al., 2013; Oh et al., 2018; Kates et al., 2020; Muthukrishnan et al., 2021; Owen et al., 2021). These studies included the analysis of sera collected in a unique biospecimen registry of 297 patients with culture-confirmed *S. aureus* osteomyelitis (AOTrauma CPP Bone Infection Registry (Kates et al., 2019)]. The results demonstrated that anti-Gmd antibody levels ranged from undetectable (<1 ng/ml) to 300 μg/ml, and the mean concentration was 21.7 μg/ml (Lee et al., 2020). We also addressed critical questions regarding the relationships between the endogenous anti-Gmd antibodies in these patients and their clinical outcome following standard of care surgery and postoperative treatment. The results showed that all patients had measurable humoral immunity against some *S. aureus* antigens, but only 20 (6.7%; p<0.0001) had basal levels of anti-Gmd antibodies (>10 ng/ml) in their serum at the time of surgery (baseline). Of these patients, 194 (65.3%) completed the 1-year follow-up and were divided into groups based on their anti-Gmd antibody level at baseline, namely, low (<1 ng/ml, n=54; 27.8%), intermediate (<10 ng/ml, n=122; 62.9%), and high (>10 ng/ml, n=18; 9.3%), and the infection control rates were 40.7%, 50.0%, and 66.7%, respectively. The incidence of adverse outcomes in these groups was 33.3%, 16.4%, and 11.1%, respectively. While high anti-Gmd titers were not the only deciding factor in infection control, as 21 out of 194 patients (10.8%) had low titers and achieved a favorable outcome at 1-year post-surgery, by assessing anti-Gmd level as a continuous variable, we found that for every 10-fold increase in concentration, there was a 60% reduction in adverse event risk (p=0.04). Furthermore, patients with low anti-Gmd titer demonstrated a highly significant 2.7-fold increased risk in adverse outcomes (p=0.008). However, a few of these patients had high titers of anti-Gmd antibodies at baseline and had adverse outcomes following surgery, which was not due to IgG4 class switching to non-functional immunoglobulin (Owen et al., 2021). Therefore, to further understand this endogenous anti-Gmd immune response, here, we describe an optimized *in vitro* assay to quantify the autolysis-neutralizing activity of anti-Gmd antibodies and the presence of neutralizing and non-neutralizing anti-Gmd antibodies in the AOTrauma CPP Bone Infection Registry.

## Materials and Methods

### Human Subjects

All human subject research was performed with informed consent under Institutional Review Board (IRB)-approved protocols (HM20009308, 20006017, and NCT01677000). Specific serum samples from the AO Trauma Clinical Priority Program (CPP) Bone Infection Registry were selected for study based on their known anti-Gmd physical titer and the patient’s clinical outcome (Kates et al., 2020).

### TPH-101 mAb

A humanized IgG1 anti-Gmd mAb derived from 1C11 was generated by transiently transfecting the heavy- and light-chain immunoglobulin genes into ExpiCHO cells as previously described (Brannan et al., 2019), and the secreted mAb was purified from the culture supernatant *via* protein-A affinity chromatography (**Supplementary Figure S1**). These quality control studies confirmed the purity of the mAb to be >99% and its specificity for native Gmd. Specificity of the GMD protein and TPH-101 antibody is further confirmed by running the Western blot assay using bacterial culture supernatant (**Supplementary Figure S1**) and in GMD protein (**Supplementary Figure S3**).

### Optimization of Cell Wall Digestion Assay

Heat-killed *Micrococcus luteus* (ATCC No. 4698; Sigma-Aldrich, Catalog # M3770-5G) was used as a substrate for recombinant His-Gmd at final concentration of 0.075% (750 μg/ml) in phosphate-buffered saline (PBS) as we previously described (Gedbjerg et al., 2013). Triton X-100 (Sigma, Catalog # T8787-250ML) was used as a substrate-solubilizing agent. Briefly, 50 μl of 200 μg/ml of Gmd was diluted twofold in 96-well plate and 50 μl of 0.15% *M. luteus* containing various concentrations of cell wall solubilizing agent Triton X-100 was added and incubated at 37°C, and OD_600_ was measured after 5, 60, and 120 min of incubation. Percentage of lysis was calculated by subtracting OD_600_ of *M. luteus* treated with various concentrations of Gmd from OD_600_ of *M. luteus* treated with Triton X-100 and dividing the product by OD_600_ of *M. luteus* treated with Triton X-100, expressing it as a percentage.

### Optimization of Neutralization of GMD by TPH-101

Purified TPH-101 (1 mg/ml) was serially diluted in PBS, and equal volume of 40 μg/ml Gmd was added in a 96-well plate and incubated at 37°C for 15 min. After incubation, equal volume of 0.15% heat-killed *M. luteus* treated with 1% Triton X-100 was added. OD_600_ was measured after 30 and 60 min of incubation at 37°C. Percentage of neutralization was calculated by subtracting OD_600_ of *M. luteus* treated with Gmd (10 μg/ml) from OD_600_ of *M. luteus* treated with Gmd neutralized by various concentrations of TPH-101 antibody and dividing the product by the OD_600_ obtained from subtracting OD_600_ of *M. luteus* treated with Gmd (10 μg/ml) from OD_600_ of *M. luteus* (bacteria only). IBT produced antibody c21D10 (IgG1 isotype; Catalog # 0200-003, Lot # 1811002) (6.643 mg/ml) was used as negative isotype control. The neutralizing concentration (NC_50_) value was determined using Sigmoidal 4PL, where X is concentration, and the least squares fit was used to quantify the 50% (NC_50_) of TPH-101 in the 30- and 60-min incubation.

### Determination of Gmd Neutralizing Human Serum Titers

Human serum samples were heat inactivated for 30 min at 56°C. Equal volume of heat-inactivated human serum samples (neat) and Gmd (40 μg/ml) was incubated at 37°C for 15 min. After incubation, equal volume of 0.15% *M. luteus* treated with 1% Triton X-100 was added. OD_600_ was measured after incubating at 37°C for 30 and 60 min, and percentage of neutralization was calculated as above.

### Spiking of Human Serum Samples

Human serum sample without physical titers against Gmd (ELISA) from the AO Clinical Priority Program (AO-CPP) cohorts were diluted 1:20, and 1 mg/ml of TPH-101 was added and incubated at 37°C with equal volume of Gmd (40 μg/ml) for 15 min. After incubation, the substrate was added, and OD_600_ was measured after 30 and 60 min. TPH-101 was used as positive control, c21D10 was used as negative control, and non-spiked serum was used as non-neutralizing control.

## Results

### Optimization of *M. luteus* Cell Wall Lysis Assay by Recombinant Gmd

**Figure 1:**
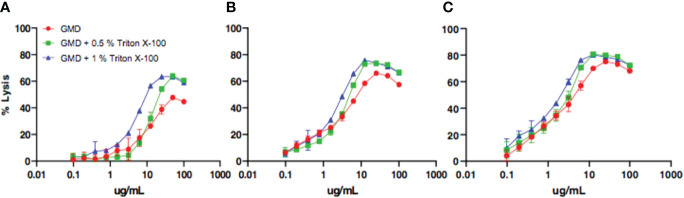
Optimization of *in vitro* assay assessing Gmd digestion of *M. luteus* cell wall. The indicated concentration (μg/ml) of purified recombinant *S. aureus* Gmd was incubated with heat-killed *M. luteus* in the presence of the indicated amount of Triton X-100 at 37°C for 5 min **(A)**, 60 min **(B)**, or 120 min **(C)**, and the percentage of lysis of *M. luteus* cell wall extract was determined by optical density as described in *Materials and Methods*. Note that the peak percentage of lysis (~80%) was achieved with a concentration of 10 μg/ml Gmd, 0.5% Triton X-100, and incubation time of 60 min.

Prior to assessing the Gmd autolysis-neutralizing activity of the patient sera (hereafter referred to as “neutralizing”), we aimed to optimize the *M. luteus* cell wall digestion assay by solubilizing the substrate in varying concentrations of a non-ionic detergent (Triton X-100). **Figure 1** shows the results of a representative experiment in which the concentration of the enzyme, detergent, and incubation time were varied to identify the condition that achieved the greatest percentage of lysis. Based on these results, we established 10 μg Gmd/ml and 0.5% Triton X-100 in a 30- and 60-min incubation period as ideal for *M. luteus* digestion.

### Neutralizing Efficiency of Humanized Anti-Gmd mAb TPH-101

**Figure 2:**
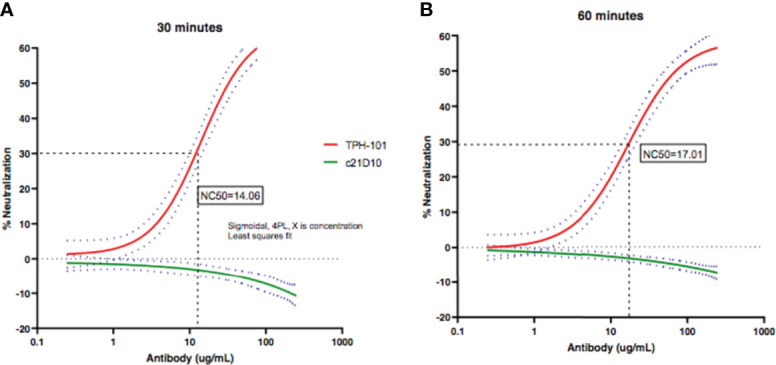
Quantification of the neutralizing activity of humanized anti-Gmd mAb (TPH-101) *in vitro*. The indicated concentration (μg/ml) of purified anti-Gmd TPH-101 mAb or irrelevant control mAb (c21D10) was added to 10 μg/ml of recombinant Gmd prior to incubation with 0.075% heat-killed *M. luteus* cell wall extract in the presence of 0.5% Triton X-100 at 37°C for 30 min **(A)** or 60 min **(B)**, and the percentage of lysis was determined as described in **Figure 1**. These data were reanalyzed using Sigmoidal, 4PL, X is concentration least squares fit to quantify the 50% neutralizing concentration (NC_50_) of TPH-101, which is 14.1 μg/ml in the 30-min incubation **(A)** and 17.0 μg/ml in the 60-min incubation **(B)**, respectively. Dotted red and green lines are ± SD.

**Figure 3:**
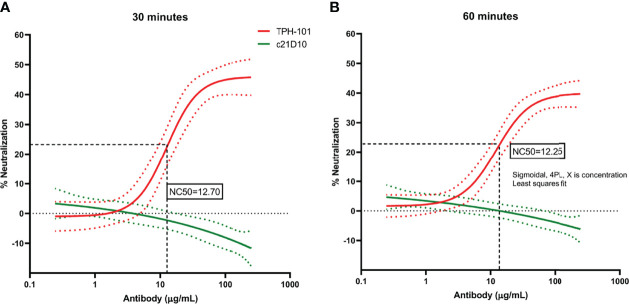
Quantification of the neutralizing activity of humanized anti-Gmd mAb (TPH-101) *via M. luteus* killing assay. The indicated concentration (μg/ml) of purified anti-Gmd TPH-101 mAb or irrelevant control mAb (c21D10) was added to 10 μg/ml of recombinant Gmd prior to incubation with live *M. luteus* in the presence of 0.5% Triton X-100 at 37°C for 30 min **(A)** or 60 min **(B)**. The percentage of neutralization was determined as described in *Material and Methods*. Sigmoidal, 4PL, X is concentration least squares fit to quantify the 50% neutralizing concentration (NC_50_) of TPH-101, which is 12.70 μg/ml in the 30-min incubation **(A)** and 12.25 μg/ml in the 60-min incubation **(B)**, respectively. Dotted red and green lines are ± SD.

To determine the concentration of purified TPH-101 required to neutralize 50% (NC_50_) of Gmd enzyme activity in the *M. luteus* cell wall lysis assay, we performed a dose–response study using the optimized *in vitro* conditions described in **Figure 1**. The results confirmed the high efficiency of TPH-101 vs. an irrelevant control IgG (c21D10), which demonstrated that the humanized anti-Gmd mAb has an NC_50_ of ~15.5 μg/ml (**Figure 2**). To confirm that this anti-Gmd neutralizing activity of TPH-101 was functional against live bacteria, we repeated this assay on cultured *M. luteus* and assessed cytolysis *via* colony-forming unit (CFU) assay, which demonstrated a similar NC_50_ of ~12.5 μg/ml (**Figure 3**). Briefly, CFU was assayed by adding 100 μl of treated ML to 900 μl of PBS and serially diluted 10-fold across 6 points (10^−1^ to 10^−6^), and 100 μl was plated on tryptic soy agar (TSA) plates, and colonies were counted by incubating at 37°C for 48 h.

**Figure 4:**
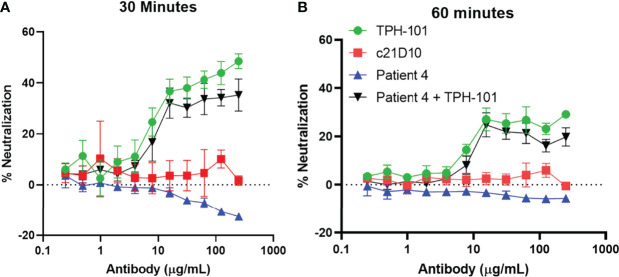
TPH-101 complementation of anti-Gmd antibody-deficient human serum *in vitro*. The indicated concentration of purified anti-Gmd TPH-101 mAb, irrelevant control mAb (c21D10), or twofold serial dilutions of human sera that do not contain neutralizing anti-Gmd antibodies (Patient 4 in the AO-CPP cohort) was added to 10 μg/ml of recombinant Gmd prior to incubation with 0.075% heat-killed *M. luteus* cell wall extract in the presence of 0.5% Triton X-100 at 37°C for 30 min **(A)** or 60 min **(B)**, and the percentage of neutralization was determined as described in **Figure 2**. Complementation of the patient 4 serum was also assessed by addition of TPH-101 or c21D10 at the indicated concentration. No differences in the percentage of neutralization of purified TPH-101 vs. TPH-101 in the human serum were detected at any concentration of antibody.

To exclude the possibility that a factor in the serum is interfering with the assay, we evaluated the efficiency of TPH-101 complementation of human sera deficient in anti-Gmd antibodies. We performed Gmd inhibition assays with purified TPH-101 and TPH-101 added to sera from patients that had no detectable titers of anti-Gmd antibodies (**Figure 4**). The results showed ~100% complementation efficiency, as no differences in the percentage of neutralization was observed at any antibody concentration. These data indicated that those sera are truly lacking anti-Gmd neutralizing activity.

### Characterizing Physical and Neutralizing Anti-Gmd Antibodies in a Select Cohort of Osteomyelitis Patients With Known Clinical Outcome

Although we have previously described the association of anti-Gmd antibody physical titers with clinical outcome of the patients in the AOTrauma CPP Bone Infection Registry (Kates et al., 2020), the Gmd neutralizing titers were unknown. Therefore, we used the *M. luteus* cell wall digestion assay to quantify the Gmd neutralization activity in a small subset of patients that (1) had high physical titers of anti-Gmd antibodies at baseline (>10 ng/ml), (2) never had detectable anti-Gmd titers throughout their treatment, or (3) developed high titers of anti-Gmd at some point during their treatment. The results are presented with the clinical outcomes in **Table 1** and contain two interesting observations. The first is that only 5 out of the 11 patients studied develop anti-Gmd neutralizing antibodies, which correlated with anti-Gmd physical titers >9,000 mean fluorescence intensity (MFI), which equates to >10 ng/ml in serum. The second observation was made by associating the patients’ antibody response with their clinical outcome over the course of treatment, which revealed that these patients can be characterized into four groups. Group 1 patients (n=3) had high physical titers and neutralizing anti-Gmd at baseline that decreased with clinical cure of the infection over time. Group 2 patients (n=3) had undetectable anti-Gmd antibodies throughout the study and adverse outcomes. Group 3 (n=3) had high titers +/− neutralizing anti-Gmd at baseline with adverse outcomes. Group 4 (n=2) had low titers of non-neutralizing anti-Gmd at baseline with delayed high titers and adverse outcomes. Collectively, these findings demonstrate that both neutralizing and non-neutralizing anti-Gmd antibodies exist in *S. aureus* osteomyelitis patients and that screening for the types of antibodies could have value for identifying patients in need of passive immunization prior to surgery.

**Table 1:** Categorical clinical outcomes and anti-Gmd responses.

Sample ID	Age	Sex	BMI	Diabetes	CCI	Clinical outcome	Gmd titer normalized to baseline	Gmd titer (MFI)	% Neutralization
**Group 1**						**High titer and neutralizing anti-Gmd at baseline that decreased with Cure**			
Patient 1	86	F	28	No	0		1	9,056.75	25
Baseline									
6 months							0.21	1,935.5	<0
12 months						Cured	0.09	780.25	<0
Patient 2	51	M	27	No	0		1	11,237.25	17
Baseline									
6 months							0.36	4,009	<0
12 months						Cured	0.14	1,529	<0
Patient 3	60	M	34	No	1		1	15,981.75	38
Baseline									
6 months							0.04	617.25	<0
12 months						Cured	0.02	242.75	<0
**Group 2**						**Undetectable anti-Gmd antibodies throughout the study and adverse outcome**			
Patient 4	60	M	30	No	1		1	284.25	<0
Baseline									
6 months							0.83	236	<0
12 months						Refractured	1.37	390	<0
Patient 5	58	M	22	No	0		1	414.75	<0
Baseline									
6 months							0.69	286.5	<0
12 months						Pseudarthrosis	0.43	179	<0
Patient 6	75	F	38	No	23		1	378	<0
Baseline									
6 months							1.15	434	<0
12 months						Reinfected @ 1 yr	0.98	369	<0
**Group 3**						**High titers of anti-Gmd at Baseline with Adverse outcome**			
Patient 7	69	M	31	No	0		1	8,356.25	<0
Baseline									
6 months							1.25	10,457.75	<0
12 months						Fistula, enterococcus	1.35	11,302	<0
Patient 8	76	M	36	No	3	Fusion knee	1	23,017.75	29
Patient 9	47	F	46	No	1	Wound breakdown	1	14,230.75	<0
**Group 4**						**Low titers of anti-Gmd at Baseline with delayed high titers and Adverse outcome**			
Patient 10	70	M	31	NR	NR		1	3,535.75	<0
Baseline									
6 months							0.93	3,283	<0
12 months						Amputation	4.35	15,382.5	<0
Patient 11	57	M	29	No	0		1	4,395	<0
Baseline									
6 months							0.23	1,028.75	<0
12 months						Nonunion, control	2.87	12,594.25	35

We were also interested to know if anti-Gmd antibody physical titers correlate with Gmd neutralizing activity in the patient sera. Thus, we performed a linear regression analysis on the five sera samples that contained Gmd neutralizing activity, and our negative findings are presented in **Supplementary Figure S2**.

## Discussion

Development of an effective immunotherapy against *S. aureus* would be transformative for orthopedic surgery and many other infections caused by this pathogen. Here, we have focused on the hypothesis that an ideal mAb would act both directly *via* antimicrobial effects through inhibition of a critical *S. aureus* target and have immunomodulatory activity to enhance the host response and bacterial clearance. From non-biased antigen discovery, *in vitro*, animal model, and clinical research, we identified Gmd as a validated target for immunotherapy (Varrone et al., 2011; Gedbjerg et al., 2013; Varrone et al., 2014; Nishitani et al., 2015; Oh et al., 2018). Based on this, we developed a lead anti-Gmd mAb (1C11) from over 36 candidates, based on its superior *in vitro* characteristics (Varrone et al., 2011; Gedbjerg et al., 2013; Varrone et al., 2014; Nishitani et al., 2020) and its safety and efficacy in animal models (Varrone et al., 2014; Yokogawa et al., 2018; Lee et al., 2020). As might be anticipated, we found that this antibody, which interferes with an enzyme expressed on the surface of the bacteria that is critical for cell wall biosynthesis, synergizes with the standard of care antibiotic therapy (vancomycin) in a one-stage exchange model of MRSA *via* distinct mechanisms of actions. Vancomycin decreased the bacterial burden on the implant, while anti-Gmd mAb inhibited *Staphylococcus* abscess communities (Yokogawa et al., 2018). We also showed feasibility of anti-Gmd mAb passive immunization by demonstrating safety and favorable pharmacokinetics following a clinically relevant dose in sheep (Lee et al., 2020).

Results from clinical research to define native humoral immunity against *S. aureus* in osteomyelitis patients also supports the hypothesis that passive immunization with anti-Gmd mAb may be an effective treatment (Gedbjerg et al., 2013; Oh et al., 2018; Kates et al., 2020; Owen et al., 2021). Most notable are the results from the AOTrauma CPP Bone Infection Registry, which showed that only 6.7% of patients with life-threatening *S. aureus* osteomyelitis have basal levels of anti-Gmd antibodies (>10 ng/ml) in their serum at the time of surgery, and that for every 10-fold increase in anti-Gmd antibody concentration in sera, there is a 60% reduction in adverse event risk (Kates et al., 2020). Furthermore, low anti-Gmd titer patients have a highly significant 2.7-fold increased risk in adverse outcomes within 1 year of surgery (Kates et al., 2020). However, in contrast to our hypothesis of passive immunization with anti-Gmd mAb, we found that a few patients had high titers of anti-Gmd antibodies at baseline and had adverse outcomes following surgery, which was not due to IgG4 class switching to non-functional immunoglobulin (Owen et al., 2021). Thus, we aimed to determine if this was due to non-neutralizing antibodies. By optimizing the *M. luteus* cell wall digestion assay and validating the neutralizing activity of TPH-101 in human sera (**Figures 1** **–** **4**), here, we show the potential of a companion diagnostic with the sensitivity and specificity necessary to assess neutralizing and non-neutralizing anti-Gmd antibodies in sera from patients. Current research is directed towards formal validation of this assay as a clinical diagnostic.

Effective humoral immunity against an infectious agent posits that high titers of neutralizing antibodies are induced, and these antibodies disappear over time after the pathogen is cleared from the host. Indeed, this is the humoral response that we observed in Group 1 patients cured of their *S. aureus* osteomyelitis and illustrates what effective anti-Gmd mAb therapy would look like (**Table 1**). Additionally, our finding that *S. aureus* osteomyelitis patients never develop neutralizing anti-Gmd antibodies (Group 2) or develop them too late in the disease process (Group 4) indirectly supports our hypothesis of anti-Gmd mAb therapy. It was also interesting to see that some patients who develop high titers of non-neutralizing antibodies also succumb to serious adverse events from *S. aureus* infection (Group 3) and that anti-Gmd physical titer does not correlate with Gmd neutralizing activity (**Supplementary Figure S2**). Taken together, these results provide the first evidence that only neutralizing antibodies are helpful in fighting off *S. aureus* bone infection and that patients who are unable to mount this specific humoral response may benefit from passive immunization with mAb like TPH-101.

As a small clinical pilot, there are several major limitations that need to be noted. In addition to the minimal numbers of patients studied, which are too small to make formal conclusions other than both neutralizing and non-neutralizing anti-Gmd antibodies existing in *S. aureus* osteomyelitis patients, our analyses were *post-hoc*. Thus, appropriately powered prospective studies of patients with (1) neutralizing anti-Gmd antibodies, (2) non-neutralizing antibodies, and (3) undetectable anti-Gmd antibodies at the time of their surgery are needed to validate the association of neutralizing antibodies with clinical outcome. It is also important to note that some clinical outcomes do not have a straightforward interpretation. For example, patient 8 had high titers of neutralizing anti-Gmd antibodies at baseline and had a knee fusion that we scored as an “Adverse” outcome based on our prospective criterion. However, this successful infection control, potentially aided by the patient’s anti-Gmd antibodies, may have been the best possible outcome based on the patient’s global health (76 years old with Class III obesity) and the damaged bone and soft tissue at the time of surgery. Finally, while the *M. luteus* cell wall digestion assay proved very useful for these research studies, we do not suggest that it can be translated into a clinical diagnostic due to the technical demands of the assay. Thus, efforts to develop a lateral flow assay to assess anti-Gmd as a biomarker are warranted.

## Data Availability Statement

The raw data supporting the conclusions of this article will be made available by the authors, without undue reservation.

## Ethics Statement

The studies involving human participants were reviewed and approved by Virginia Commonwealth University HRPP. The patients/participants provided their written informed consent to participate in this study. The animal study was reviewed and approved by University of Rochester.

## Author Contributions

All authors were directly involved in designing the experiments, data analysis, and drafting the manuscript. SS, GM, TK, and JO performed experiments. SK is the principal investigator of the IRB-approved clinical research. All authors contributed to the article and approved the submitted version.

## Funding

This work was supported by research grants from the National Institutes of Health (R44 AI155309, P30 AR69655 and P50 AR72000, and NCATS 1UL1TR002649) and the AOTrauma Clinical Priority Program.

## Conflict of Interest

SS, RA, TK, and MA are paid employees of Integrated Biotherapeutics Inc. RP and ES are paid consultants of Integrated Biotherapeutics Inc. and have stock in Telephus, LLC.

The remaining authors declare that the research was conducted in the absence of any commercial or financial relationships that could be construed as a potential conflict of interest.

## Publisher’s Note

All claims expressed in this article are solely those of the authors and do not necessarily represent those of their affiliated organizations, or those of the publisher, the editors and the reviewers. Any product that may be evaluated in this article, or claim that may be made by its manufacturer, is not guaranteed or endorsed by the publisher.

## Supplementary Material

Supplementary Figure S1 Quality controls of purified TPH-101 anti-Gmd mAb. ExpiCHO cells were transiently transfected with TPH-101 heavy and light chain genes, and the secreted mAb was purified from culture supernatant *via* protein-A affinity chromatography as previously described (Brannan et al., 2019). The total yield of TPH-101 mAb was 400 ug/ml, and the purity was determined to be >99% *via* Coomassie-stained denatured SDS-PAGE and SEC-HPLC chromatograph. No degradation of the mAb was observed. The specificity of TPH-101 binding to Gmd was confirmed *via* western blot of total *S. aureus* USA300 protein extract as previously described (Varrone et al., 2014).

Supplementary Figure S2 Lack of correlation between anti-Gmd antibody physical IgG titer and Gmd neutralizing activity in human sera. A linear regression analysis of anti-Gmd physical IgG titer determined by Luminex vs. Gmd neutralizing activity determined by *M. luteus* cell wall digestion was performed on the five patient sera that contained Gmd neutralizing activity described in **Table 1**. No significant association was found by the Spearman’s rank correlation coefficient.

Supplementary Figure S3 Gmd protein is specifically detected by TPH-101 antibody in Western Blot assay. 100 and 50 ng of recombinant Gmd protein was heated at 70°C for 10 min and separated *via* SDS-PAGE. Gel incubated with 1 µg/ml anti-THP 101 (lot# 2378-1A-02) for 2 hr and detected by 1:3000 dilution of KPL Goat anti-Human HRP (KPL#474-1006; lot#150100) for 1 hr using chemiluminescence.
