# Supplementary figures and images for: Evidence of Neutralizing and Non-Neutralizing Anti-Glucosaminidase Antibodies in Patients With S. Aureus Osteomyelitis and Their Association With Clinical Outcome Following Surgery in a Clinical Pilot
Source: Front Cell Infect Microbiol. 2022 Jul 18;12:876898. doi: 10.3389/fcimb.2022.876898 (PMC9339635; doi:10.3389/fcimb.2022.876898)

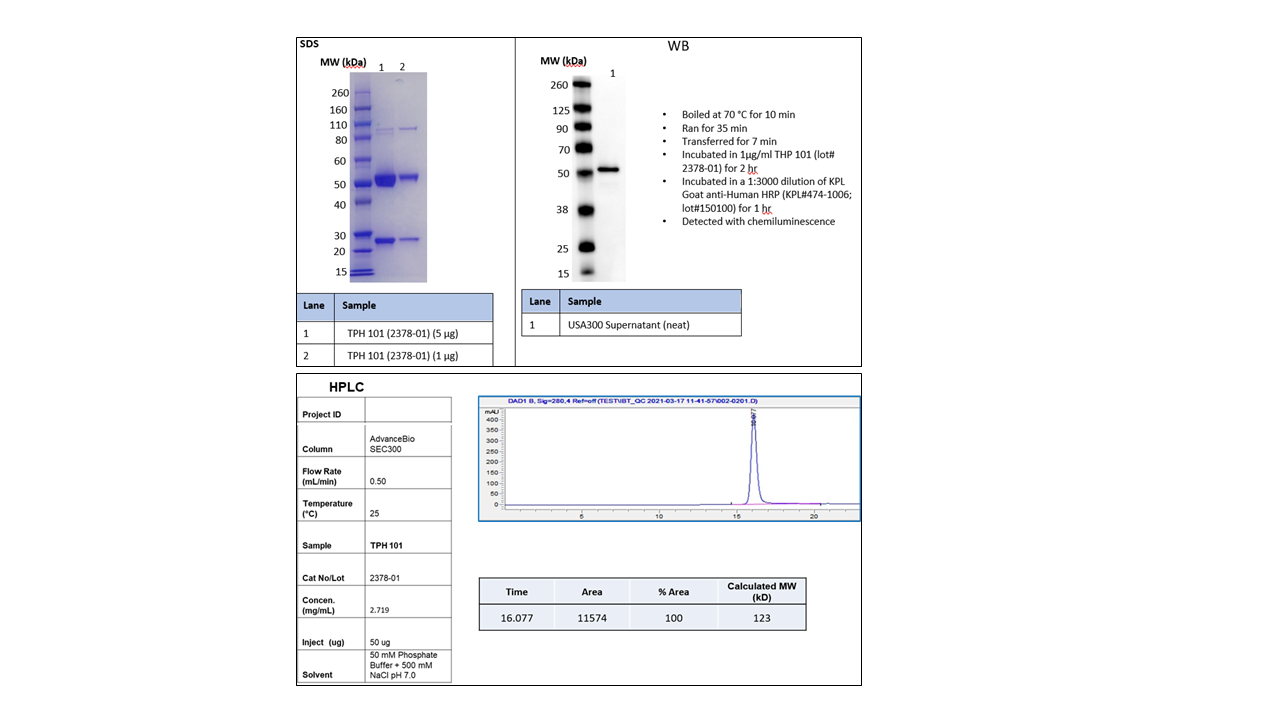

Supplement: Supplementary Figure S1 — Quality controls of purified TPH-101 anti-Gmd mAb. ExpiCHO cells were transiently transfected with TPH-101 heavy and light chain genes, and the secreted mAb was purified from culture supernatant via protein-A affinity chromatography as previously described (Brannan et al., 2019). The total yield of TPH-101 mAb was 400 ug/ml, and the purity was determined to be >99% via Coomassie-stained denatured SDS-PAGE and SEC-HPLC chromatograph. No degradation of the mAb was observed. The specificity of TPH-101 binding to Gmd was confirmed via western blot of total S. aureus USA300 protein extract as previously described (Varrone et al., 2014). [file Image_1.tif]

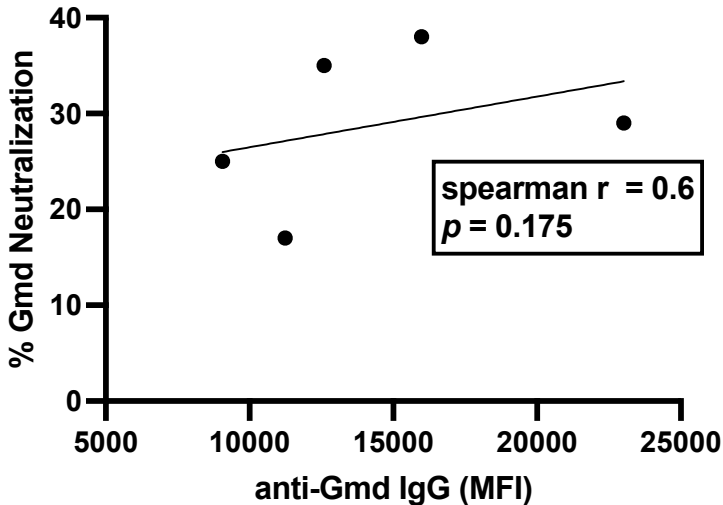

Supplement: Supplementary Figure S2 — Lack of correlation between anti-Gmd antibody physical IgG titer and Gmd neutralizing activity in human sera. A linear regression analysis of anti-Gmd physical IgG titer determined by Luminex vs. Gmd neutralizing activity determined by M. luteus cell wall digestion was performed on the five patient sera that contained Gmd neutralizing activity described in Table 1 . No significant association was found by the Spearman’s rank correlation coefficient. [file Image_2.pdf]

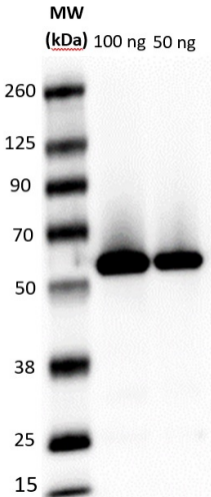

Supplement: Supplementary Figure S3 — Gmd protein is specifically detected by TPH-101 antibody in Western Blot assay. 100 and 50 ng of recombinant Gmd protein was heated at 70°C for 10 min and separated via SDS-PAGE. Gel incubated with 1 µg/ml anti-THP 101 (lot# 2378-1A-02) for 2 hr and detected by 1:3000 dilution of KPL Goat anti-Human HRP (KPL#474-1006; lot#150100) for 1 hr using chemiluminescence. [file Image_3.pdf]
